# Processing of Viral DNA Ends Channels the HIV-1 Integration Reaction to Concerted Integration[Fn FN1]

**DOI:** 10.1074/jbc.M505367200

**Published:** 2005-06-14

**Authors:** Min Li, Robert Craigie

**Affiliations:** Laboratory of Molecular Biology, NIDDK, National Institutes of Health, Bethesda, Maryland 20892

## Abstract

Retroviral DNA made by reverse transcription is blunt-ended, and the viral integrase protein must remove two nucleotides from each 3′ end prior to integration into chromosomal DNA. Under most reaction conditions for integration *in vitro*, the majority of the reaction products are “half-site” products that result from integration of only one viral DNA end into one strand of the target DNA. Preprocessed DNA substrates are more efficient substrates for half-site reactions than are blunt-ended substrates, which require the removal of two nucleotides prior to integration. In contrast, we find that blunt-ended DNA is a better substrate for the biologically relevant reaction of concerted integration of pairs of viral DNA ends. The reaction pathway is channeled to concerted integration, and half-site integration products are reduced with blunt-ended DNA substrate that must first be processed by integrase. In addition, the terminal nucleotide requirements for concerted integration are more stringent than for the half-site reaction. Longer DNA is more efficient for the concerted reaction than is shorter DNA that is capable of efficient half-site integration. This suggests that nonspecific interactions of integrase with viral DNA distant from the termini contribute to the assembly of a complex that is competent for concerted integration. Finally, differential effects of mutation of a residue in the C-terminal domain of integrase on concerted *versus* half-site integration implicate protein-protein interactions involving this domain as important for concerted integration.

Integration of retroviral DNA into the host chromosomal DNA, an essential step in the retroviral replication cycle, involves two chemical steps (1). The newly synthesized blunt-ended viral DNA first undergoes 3′ end processing. In this reaction, two nucleotides are removed from each 3′ end. Next, the exposed hydroxyl groups attack a pair of phosphodiester bonds on opposite strands of the target DNA to complete the strand transfer step. For human immunodeficiency virus (HIV)-1,^1^ the sites of attack are separated by 5 bp, resulting in a five-base duplication of target DNA sequence upon repair of the resulting integration intermediate. In the presence of a divalent metal ion, the viral integrase protein alone is able to carry out both 3′ processing and DNA strand transfer with simple DNA substrates that mimic the viral DNA ends (2–4). However, under most reaction conditions the products of DNA strand transfer result from the integration of a single viral DNA end into one strand of the target DNA, a reaction that has been termed “half-site” as opposed to concerted integration. Were this to occur in the cell, the viral DNA would fail to integrate, and the viral replication cycle would be aborted.

Following reverse transcription, the viral DNA remains associated with integrase and other viral and cellular proteins as part of the preintegration complex (PIC) (5–13). PICs isolated from infected cells efficiently integrate their DNA into a target DNA *in vitro*. In contrast to the typical reaction products with purified integrase, the products mostly result from concerted integration of a pair of viral DNA ends into target DNA. Although early work demonstrated that the integrase proteins of several retroviruses could accomplish concerted integration, the efficiency was extremely low and required genetic selection to detect the products (2, 5, 14, 15). More recently, Grandgenett and co-workers (15–22) have demonstrated that, under appropriate reaction conditions, both Rous sarcoma virus and HIV-1 integrase proteins alone are capable of much higher efficiencies of concerted integration allowing the products to be detected directly by physical assays. Other studies have suggested that viral or cellular proteins in addition to integrase may be involved in promoting concerted integration (23–26).

In principle, biochemical dissection of the preintegration complex could reveal the factors that contribute to highly efficient concerted integration. Unfortunately, these complexes are present in low abundance in cell extracts, and our knowledge of even their protein composition alone is largely limited to that obtainable from immunoprecipitation experiments. Because PICs are not readily amenable to detailed biochemical analysis at the molecular level, it is necessary to reconstitute complexes with all the features of those isolated from cells to fully understand their functioning. To this end, we have investigated the factors that promote concerted integration by HIV-1 integrase. We found that blunt-ended DNA substrates are more efficient for concerted integration than “preprocessed” substrates. This indicated that the 3′ processing reaction pathway plays a role in channeling the reaction to the concerted integration pathway. In addition, the 2-base overhang-generated 3′ processing is important for promoting concerted integration. These findings suggest a rationale for why HIV-1 synthesizes an additional two nucleotides beyond the proviral sequence; the 2-base overhang generated by 3′ processing is important for the subsequent integration step. A gain of function mutant in the C-terminal domain of integrase was found to carry out more efficient concerted integration than the wild type protein, whereas a different mutation of the same residue abolished concerted integration without affecting the half-site reaction. These results suggest that the C-terminal domain is involved in a multimerization interface that is required for concerted integration but is dispensable for the half-site reaction.

## MATERIALS AND METHODS

### DNA Substrates—

The 355-bp linear mini-viral DNAs were prepared by NdeI or ScaI digestion of pNde355 and pSca355, respectively, and purified on agarose gels. NdeI or ScaI digestion of these plasmids, which are based on pCR2.1 (Invitrogen), results in a linear fragment flanked by 21 bp of HIV-1 NL4–3 U5 LTR and U3 LTR sequences. NdeI digestion of pNde355 gives the precleaved substrate, and ScaI digestion of pSca355 gives the blunt-ended substrate. The precleaved DNA contains unique BamHI and AatII restriction sites, 61 and 248 bp from the U3 terminus, respectively. The blunt-ended DNA substrate contains unique ClaI and AatII restriction sites, 55 bp and 250 bp from the U3 terminus, respectively. The DNA fragments were treated with alkaline phosphatase before 5′ end-labeling with [*γ*−^32^P]ATP by T4 polynucleotide kinase. Target DNAs were supercoiled *ϕ*X174 (5386 bp) or pBR322 (4361 bp). The ~410-bp substrates S1, S2, and S3 were made by ligation of oligonucleotides containing 21 bp of U5 terminal sequence to the EcoRI site of a 380-bp BamH1 to EcoRI restriction fragment of pBR322. The oligonucleotides were designed so that S1 terminated with the authentic sequence of HIV-1-preprocessed DNA, S2 terminated with the authentic sequence of blunt-ended DNA, and S3 terminated with the sequence corresponding to preprocessed substrate made by NdeI cleavage.

### Protein Expression and Purification—

Except as noted, HIV-1 integrase carrying the F185H and C280S mutations was used. These mutations improve the solubility of the protein and do not compromise viral replication *in vivo* (27, 28). F185H/C280S integrase was expressed in *Escherichia coli* BL21(DE3) and purified by nickel affinity chromatography essentially as described for the catalytic domain (29). After removal of the His tag with thrombin, the protein was loaded onto a Mono S HR 10/10 column (Amersham Biosciences) and eluted with a linear gradient of 0.15–0.65 m NaCl, containing 25 mm MES, pH 6.0, 1 m urea, 1 mm EDTA, pH 8.0, 1 mm dithiothreitol, and 10% (w/v) glycerol. The peak fractions were pooled and dialyzed overnight against 1 m NaCl, 20 mm Hepes, pH 7.5, 1 mm EDTA, 1 mm dithiothreitol, and 10% (w/v) glycerol. The purified protein was concentrated using a Centriprep (YM-10 membrane, Millipore) membrane, frozen in liquid nitrogen, and stored at −80 °C. Integrase protein with the W235F or W235A mutation was purified as described for wild type His-tagged integrase (30). Briefly, the integrase was expressed in *E. coli* BL21(DE3), and the cells were lysed in buffer containing 0.1 m NaCl. The lysate was centrifuged, and integrase was extracted from the pellet in buffer containing 2 m NaCl. The protein was then purified by nickel affinity chromatography, and the His tag was removed with thrombin. HMGA1 was purified as described elsewhere (31). HMGB1 was a gift from Dr. Martin Gellert, National Institutes of Health. HIV-1 NC was a gift from Dr. Louis Henderson, NCI-Frederick, National Institutes of Health.

### Integration Assay—

Typical reaction mixtures (50-*μ*l final volume) were assembled by incubating 80 nm integrase on ice in 20 mm Hepes, pH 7.5, 12% Me_2_SO, 5 mm dithiothreitol, 10% polyethylene glycol-6000, 10 mm MgCl_2_, 20 *μ*m ZnCl_2_, and 100 mm NaCl (final) followed by the addition of 10 nm donor DNA substrate. These components were preincubated on ice for 1 h, and 500 ng of target plasmid DNA was then added. After an additional 1-h preincubation on ice, the reaction was initiated by a transfer to incubation at 37 °C, which continued for 1 h. The reactions were stopped by the addition of SDS and EDTA to 0.1% and 10 mm, respectively, together with 5 *μ*g of proteinase K. Incubation was continued at 37 °C for a further 1 h. 10 *μ*l of the reaction mixture was then electrophoresed in a 0.8% agarose gel in Tris borate-EDTA buffer. Scale-up reactions for restriction analysis or gel isolation of products were extracted with an equal volume of phenol-chloroform-isoamylalcohol and then with chloroform only followed by ethanol precipitation. Gels were dried, exposed to imaging plates, and visualized and quantified with a PhosphorImager (Amersham Biosciences).

## RESULTS

### In Vitro Integration Assay—

**Fig. 1.**
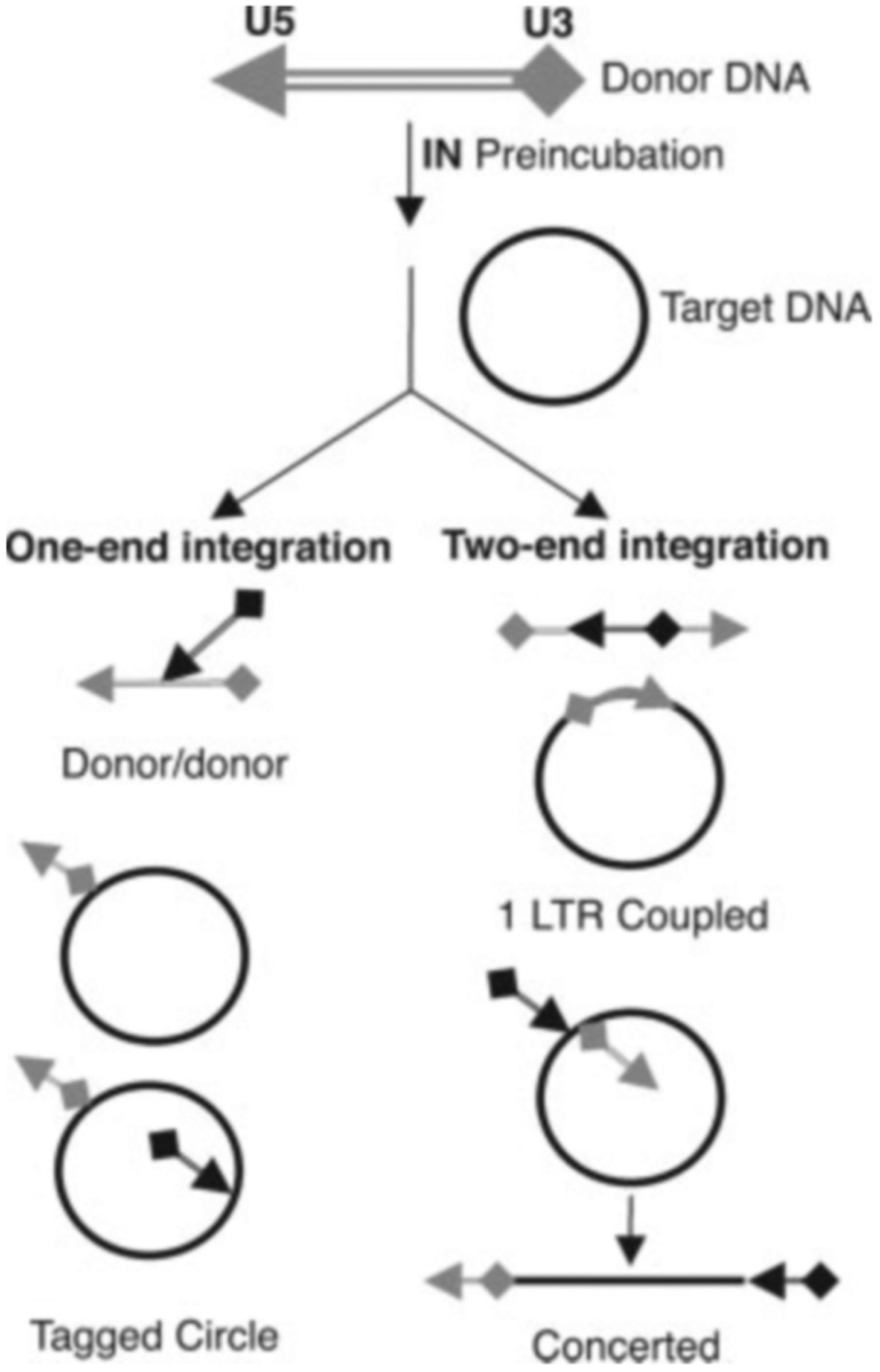
Schematic of the possible products of integration with a linear donor DNA flanked by U5 and U3 viral DNA ends and a circular target DNA. Half-site integration products are shown on the *left*, and products resulting from concerted integration of pairs of ends are shown on the *right*.

**Fig. 2.**
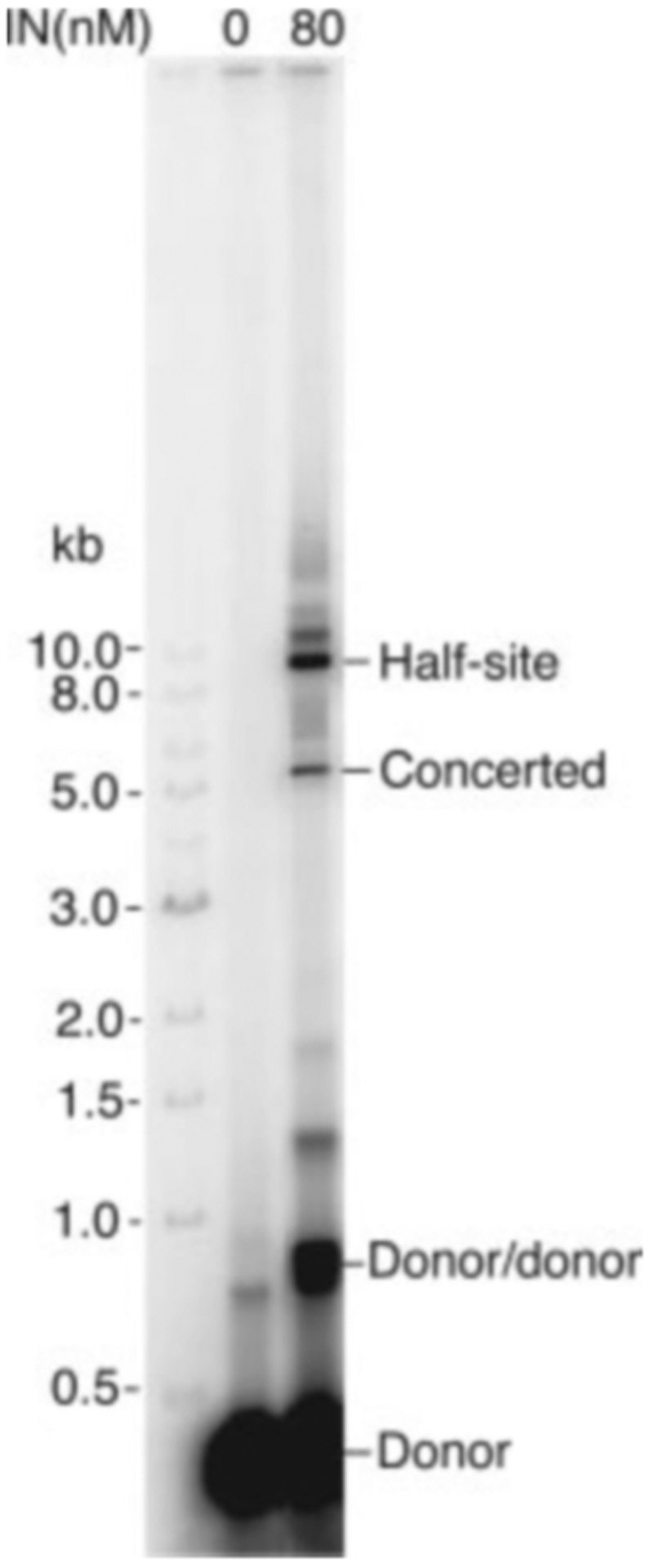
Products of an integration reaction with 355-bp U3/U5 viral DNA substrate and circular pBR322 as the target. The viral DNA was 5′ end-labeled with ^32^P. Reaction products were separated by agarose gel electrophoresis and visualized using a PhosphorImager. The positions of the 2-LTR concerted integration product, the half-site product, and products resulting from integration of viral DNA substrate into itself (*Donor/donor*) are indicated. The migration positions of linear size markers are shown on the *left*.

We initially used a 355-bp linear DNA with 21 bp of U5 LTR sequence at one end and 21 bp of U3 LTR sequence at the other end as the viral DNA substrate for integration. The ends were generated by cleavage with NdeI, which leaves the 3′ ends terminating with the CA dinucleotide corresponding to the product of 3′ end processing. Like authentic viral DNA, this substrate has a 2-base overhang at the 5′ ends, but this overhang differs in sequence from the natural substrate. Supercoiled pBR322 or *ϕ*X174 served as the target DNA for integration. The potential products of one-end or two-end integration events are depicted in Fig. 1. In preliminary experiments we explored a wide range of reaction conditions (data not shown). As reported previously by Grandgenett and co-workers (20), the presence of polyethylene glycol in the reaction mixture was found to be critical for promoting the two-end integration reaction. The products of a typical reaction are shown in Fig. 2. The identity of each band was confirmed by restriction analysis (supplemental Fig. 1). The major product of concerted integration corresponds to the product labeled concerted in Fig. 1; restriction analysis showed that, as reported by Grandgenett and co-workers (20), pairs of U5 ends were preferentially used compared with U3/U5 pairs or U3/U3 pairs (supplemental Fig. 1). Cloning and sequencing of integration products demonstrated that most exhibited the 5-bp target site duplication characteristic of HIV-1 DNA integration (supplemental Table I). The 1-LTR coupled product was not observed, probably because of the stiffness of the short DNA that energetically disfavors intramolecular juxtaposition of two ends on the same DNA molecule. The major half-site reaction products corresponded to the tagged circle and donor/donor products depicted in Fig. 1.

### Concerted Integration Is Less Sensitive to High Ionic Strength than Is Half-site Integration—

**Fig. 3.**
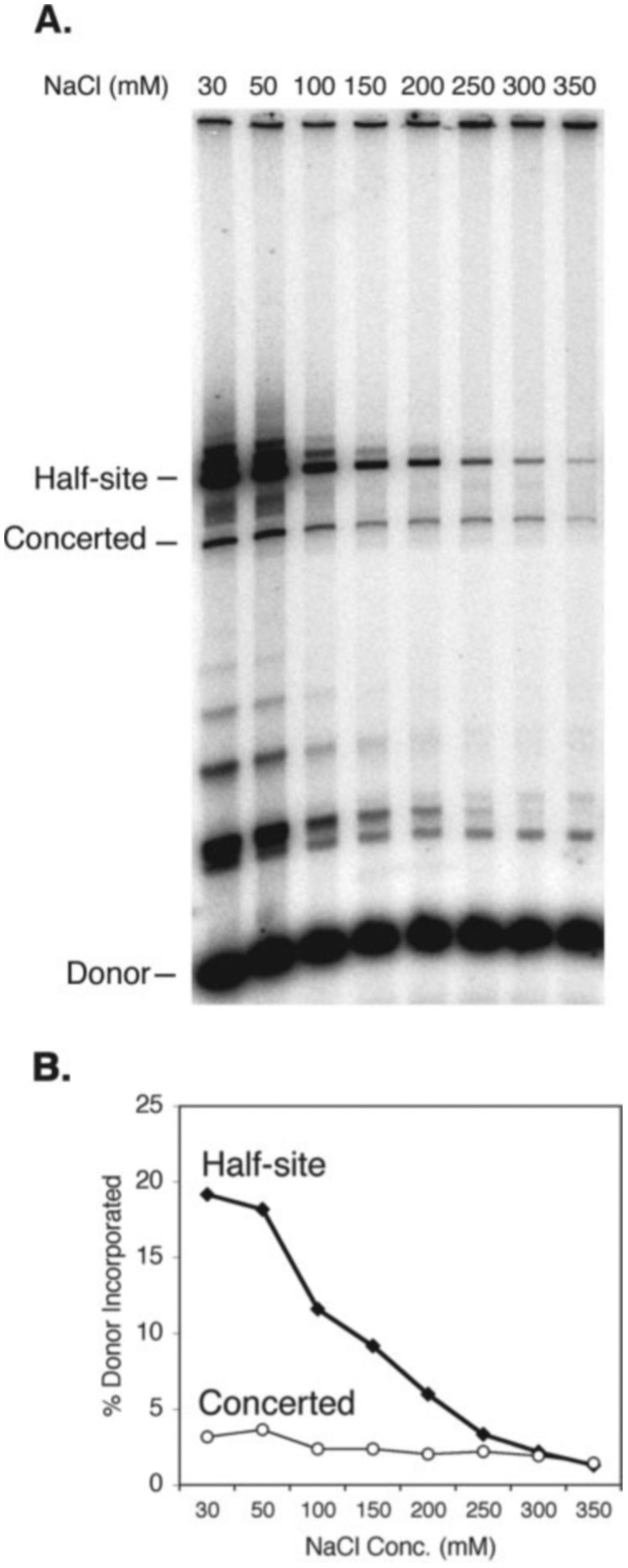
Salt dependence of concerted and half-site integration. Reaction mixtures contained preprocessed viral DNA substrate made by NdeI digestion and the indicated concentrations of NaCl. *A*, reaction products were visualized by exposure to an imaging plate. *B*, quantitation of the data shown in *A*. Half-site integration is most efficient at low salt and becomes markedly less efficient above 150 mm NaCl. In contrast, the efficiency of concerted integration is less sensitive to salt concentration. *Conc*., concentration.

Most of the literature on HIV-1 integrase activities reports assays and conditions under which the integration products almost exclusively result from half-site integration events. These assays are highly sensitive to salt, and low salt is essential for robust activity. Fig. 3 shows that in the assay reported here low salt also greatly stimulates the half-site reaction. Although the two-end reaction exhibits a similar trend, there is a much lesser salt dependence than for the half-site reaction. Although the efficiency of the half-site reaction progressively decreases with increasing ionic strength, the efficiency of concerted integration remains constant between 150 and 350 mm NaCl. The nucleoprotein complexes that mediate concerted integration are therefore more stable with high ionic strength than those that carry out the half-site reaction. This property mirrors the functional association of integrase with the preintegration complex, which is not dissociated even at greater than 0.5 m concentrations of NaCl.

### Concerted Integration Is Not Stimulated by Host Factors—

**Fig. 4.**
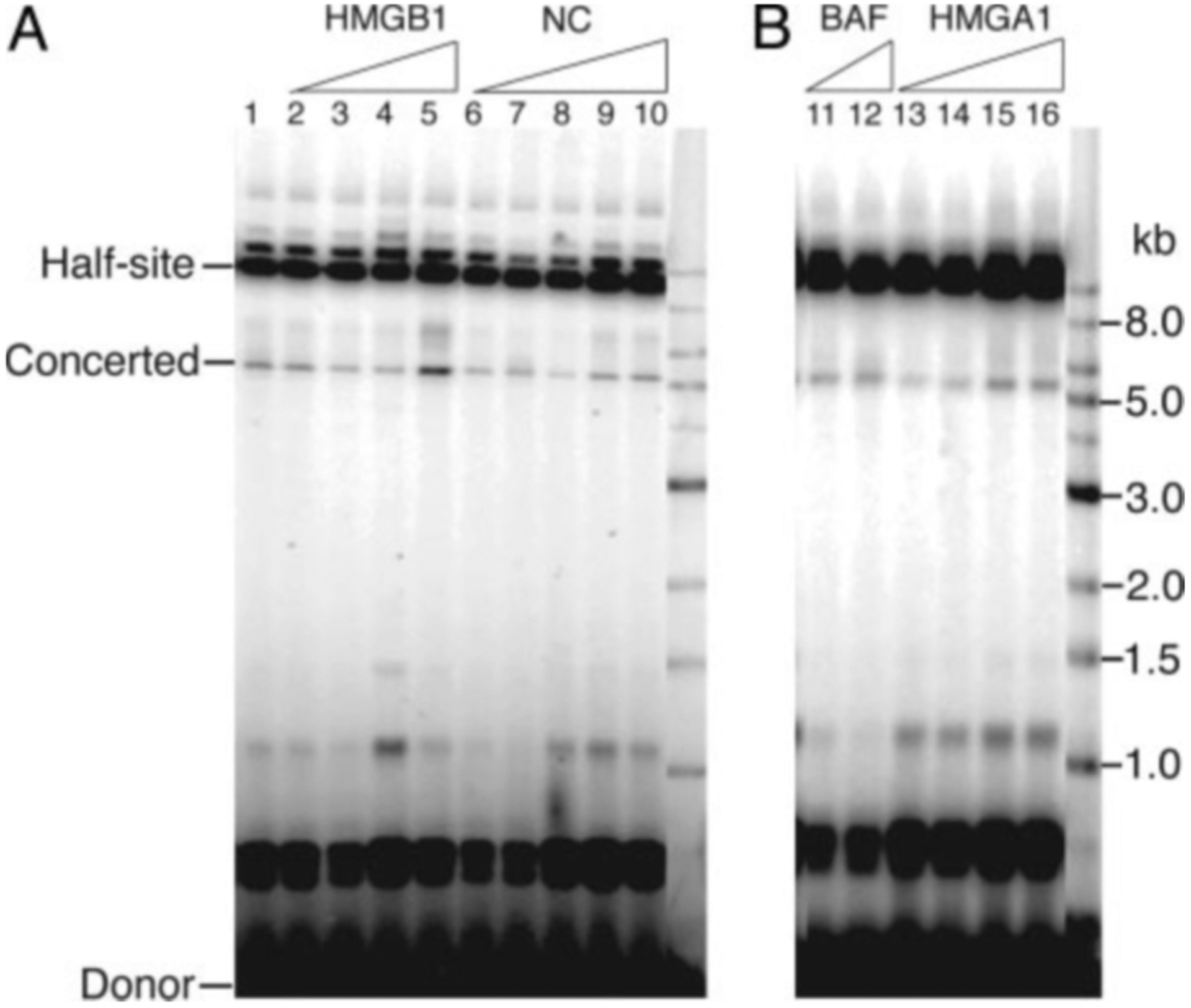
Effect of HMGB1, NC, BAF, and HMGA1 on integration activity. *Lane 1* shows the products of a reaction including only IN as a protein factor (*A*). Additional proteins were included in the integration mixture at the following concentrations: HMGB1, 40 nm, 80 nm, 320 nm, and 1.6 *μ*m (*A*, *lanes 2–5*); NC, 50 nm, 100 nm, 250 nm, 500 nm, and 2 mm (*A*, *lanes 6–10*); BAF, 50 nm and 100 nm (*B*, *lanes 11* and *12*); and HMGA1, 70 nm, 140 nm, 350 nm, and 1.4 *μ*m (*B*, *lanes 13–16*).

The Grandgenett laboratory has reported that relatively efficient concerted integration by HIV-1 integrase can occur without cellular or viral cofactors (20), whereas other reports have implicated the cellular proteins HMGB1 (23, 26) and HMGA1 (26) and the viral protein NC (25) as cofactors for concerted integration. Although they are clearly not essential, we wished to determine whether they have any effect on the efficiency of concerted integration under our assay conditions. Stimulation was observed at only the highest concentration of HMGB1, and no stimulation was observed with the other proteins tested (Fig. 4).

### Blunt-ended DNA Substrate Is More Efficient than Preprocessed Substrate for Concerted Integration—

**Fig. 5.**
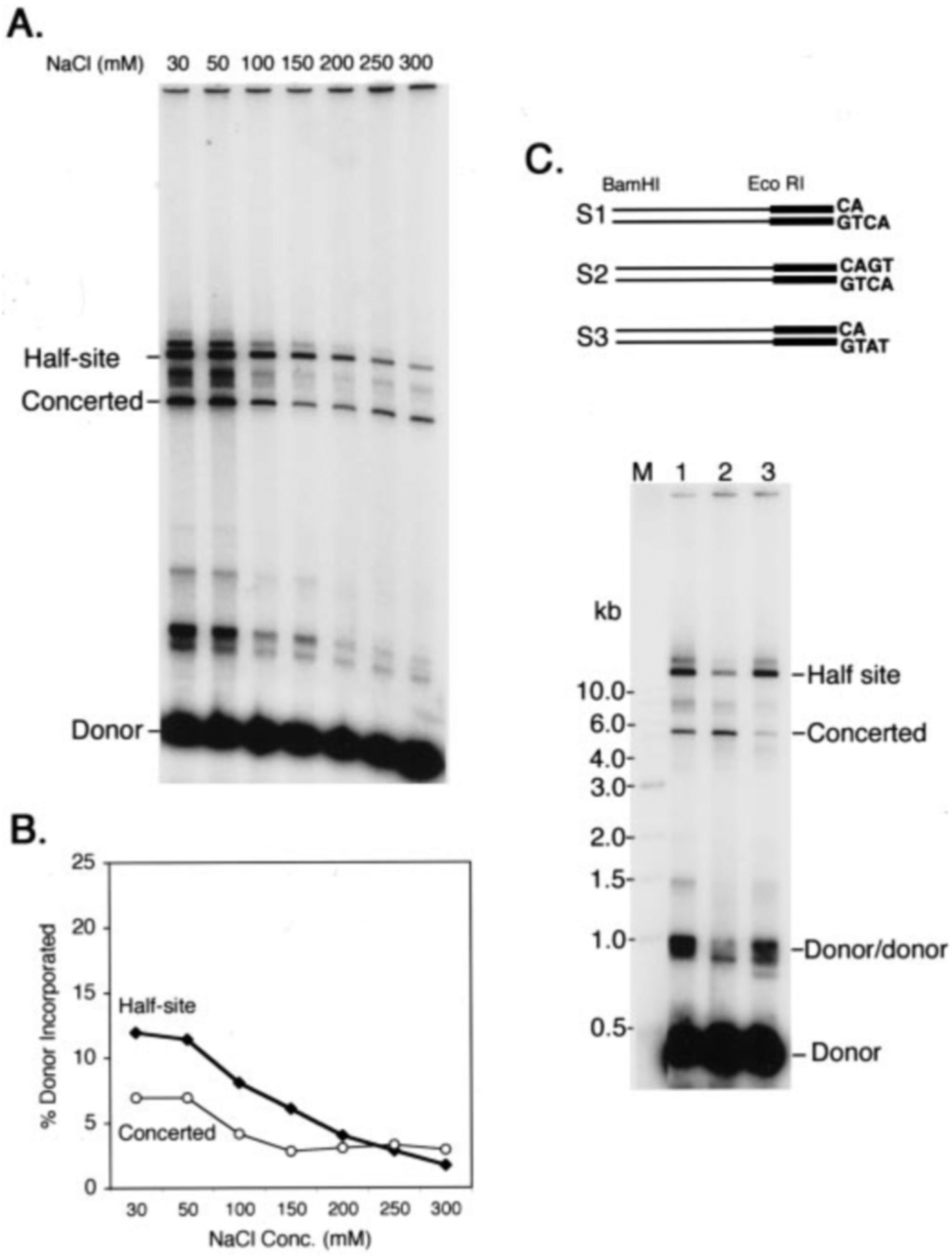
Blunt-ended viral DNA substrate favors concerted integration. *A*, reaction mixtures contained blunt-ended viral DNA substrate made by ScaI digestion and the indicated concentrations of NaCl. Products were visualized by exposure to an imaging plate (compare with Fig. 3). *B*. quantitation of the data shown in *A*. *Conc*., concentration. *C*, 410-bp substrates S1 (preprocessed with authentic terminal sequence), S2 (blunt-ended with authentic terminal sequence), and S3 (preprocessed with a terminus corresponding to that produced by NdeI cleavage) were made as described under “Materials and Methods.” Reactions contained the following DNA substrates: *Lane 1*, S1; *lane 2*, S2; *lane 3*, S3. *Lane M*, size markers.

Viral DNA made by reverse transcription is blunt-ended and must be processed to remove two nucleotides from the 3′ ends prior to the strand transfer step. However, most previous studies of concerted integration *in vitro* used preprocessed viral DNA substrates that bypass the 3′ end-processing step of the integration reaction. The issue of whether processing by integrase influences the subsequent DNA strand transfer step has not been addressed. Both blunt-ended and preprocessed substrate work efficiently in simple *in vitro* assays of DNA strand transfer that monitor the half-site reaction with short DNA substrates mimicking one end of the viral DNA. Because the concerted integration of blunt substrate requires that both ends first be processed, we anticipated that preprocessed substrate would be more efficient for concerted integration than blunt-ended substrate would be. Fig. 5 shows that, contrary to this expectation, blunt-ended substrate is actually better for concerted integration than preprocessed substrate (compare with the preprocessed substrate Fig. 3). Half-site integration events are also decreased with blunt-ended substrate.

NdeI-cleavage provides a convenient way to generate a preprocessed substrate, but the resulting 2-base overhang differs in sequence from the natural substrate. To test whether the identity of these bases influences concerted integration, we synthesized blunt-ended and preprocessed substrates with the natural terminal sequence, together with a preprocessed substrate with a terminus corresponding to the product of NdeI cleavage. Fig. 5C shows that blunt-ended DNA is more efficient for concerted integration even when compared with preprocessed DNA with the authentic terminal sequence. However, although the NdeI-cut and authentic preprocessed substrates are equally competent for half-site integration, the NdeI-cut preprocessed substrate is less efficient for concerted integration than is the authentic preprocessed substrate. The sequence of the two terminal bases therefore influences the efficiency of concerted integration; preprocessed substrates with CA and AT overhangs are equally efficient for half-site integration.

### IN/W235F Carries Out Concerted Integration More Efficiently than Wild Type IN—

**Fig. 6.**
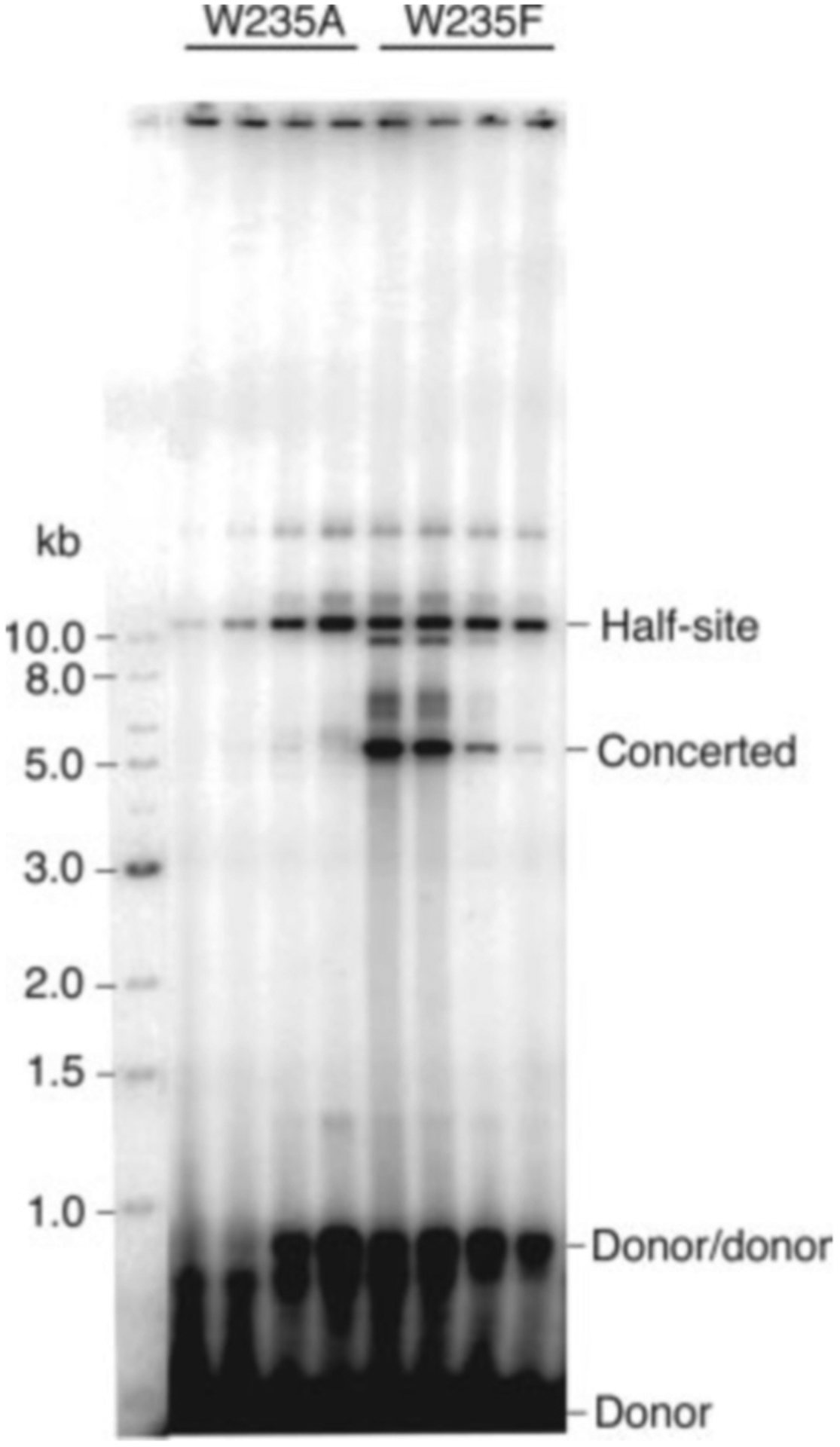
Mutation of Trp-235 differentially affects half-site and concerted integration. Reaction mixtures contained the W235A or W235F IN proteins at 40 nm (*lanes 1* and *5*), 80 nm (*lanes 2* and *6*), 200 nm (*lanes 3* and *7*), and 400 nm (*lanes 4* and *8*). W235A catalyzes half-site integration but not concerted integration (*lanes 1–4*). W235F has enhanced activity for concerted integration (*lanes 5–8*).

The W235E or W235A mutation in IN abolishes viral replication of HIV-1 even though the activity of integrase is normal in *in vitro* assays that do not distinguish concerted from half-site integration products (27, 32–34). In contrast, HIV-1 IN with the mutation W235F is replication-competent (35). We therefore tested whether the replication defect of the Trp-235 mutant might be due to an inability to carry out concerted integration. Fig. 6 shows that this is indeed the case. Half-site reaction products are seen at normal levels, whereas concerted integration products are not detected. Interestingly, the W235F mutant carried out concerted integration more efficiently than either wild type integrase (data not shown) or the F185H/C280S mutant. The profound differential effects of mutation of this residue on half-site *versus* concerted integration suggest a role in a protein-protein interface that is required for the concerted reaction. It is noteworthy that Trp-233 of Rous sarcoma virus integrase, which corresponds to Trp-235 of HIV-1 integrase, is also critical for integration. However, the phenotype of the mutants differ from those reported here for HIV-1 integrase. The Rous sarcoma virus W233A integrase is inactive for both concerted and half-site integration, whereas the W233F protein is wild type for concerted integration and slightly hyperactive for the half-site reaction (17).

### Longer Viral DNA Substrates Are More Efficient for Concerted Integration—

**Fig. 7.**
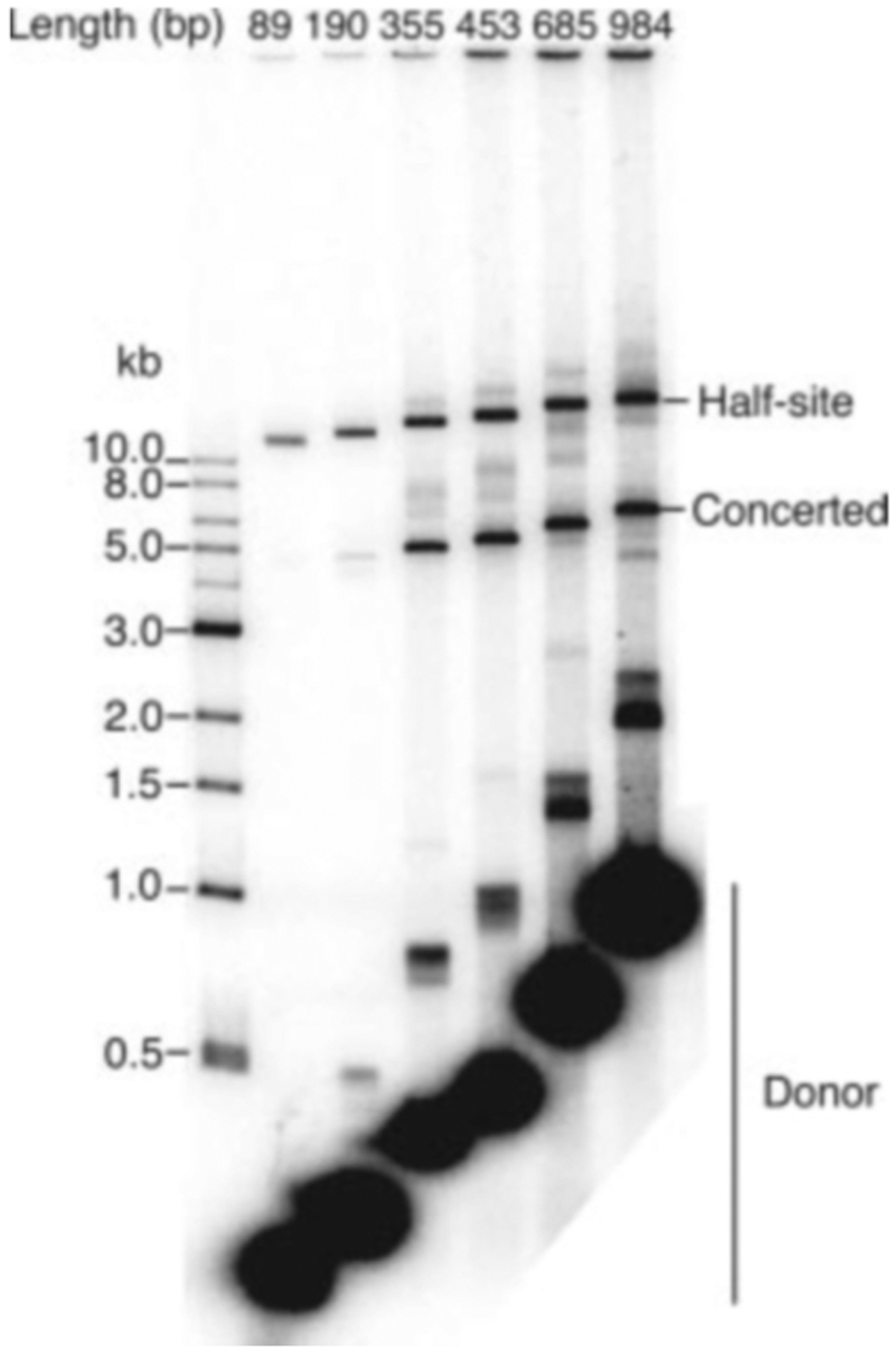
Effect of viral DNA substrate length on the efficiency of integration. Substrates contained 21 bp of blunt-ended U5 viral terminal sequence and various lengths of nonspecific DNA. The total length of each substrate is indicated at the *top* of each *lane*. Quantitation of the concerted and half-site products revealed that, over the range of DNA length shown, the amount of concerted product increased by over 40-fold, whereas the half-site product increased by only 3-fold.

20 bp of terminal viral DNA sequence are efficient substrates for half-site integration *in vitro*, but the effect of the length of the flanking DNA sequence has not been determined for the concerted reaction. We therefore constructed a set of viral DNA substrates of various lengths to test the length dependence of concerted integration. Each substrate had 21 bp of blunt-ended U5 terminal sequence at one end and a varying length of nonspecific DNA. Fig. 7 shows that several hundred base pairs are required for maximal efficiency of concerted integration, and very little concerted product was observed with substrates shorter than 200 bp. This contrasts with half-site integration, which occurs efficiently with oligonucleotide substrates as short as 20 bp (36).

## DISCUSSION

The DNA cutting and joining steps involved in retroviral DNA integration must be carefully orchestrated to ensure the proper outcome, insertion of the viral DNA into the genome of the host cell. In particular, the cleavage and joining reactions at the two ends of the viral DNA must be coordinated. Our results demonstrate that the processing of the viral DNA by integrase directs the reaction pathway toward concerted integration and away from the half-site reaction pathway. Changing the 2-base overhang on the nontransferred strand from CA to AT reduces the efficiency of concerted integration without compromising the half-site reaction. We infer that processing by integrase facilitates the formation of a synaptic complex that is competent for concerted integration and the nature of the 2-base overhang is important for the formation or stability of this complex. The importance of this 2-base overhang for concerted integration may explain why HIV-1 synthesizes an additional two nucleotides beyond the proviral sequence. We note that in the closely related Mu transition reaction, the flanking DNA on the nontransferred strand is also important for assembly of a stable synaptic complex of a pair of Mu DNA ends with transposase (37).

Although, half-site integration reactions are robust and efficient under a wide range of reaction conditions, the efficiency of concerted integration is highly sensitive to many variables, including the concentration and stoichiometry of reaction components and the presence of additives such as polyethylene glycol. This may account for differing reports in the literature on what factors are important for concerted integration. Are cellular proteins involved in concerted integration? Grandgenett and co-workers (20) have demonstrated that HIV-1 integrase alone is sufficient to carry out concerted integration. However, because a significant fraction of integration products result from half-site integration, the possibility remains that cellular or viral factors may improve the fidelity of the reaction. We therefore tested the effect of several proteins that have been shown to be a component of the PIC or implicated in promoting concerted integration. NC, HMGA1, and BAF had no effect on the efficiency of concerted integration in our system. HMGB1 showed a stimulatory effect only at the highest concentration tested, a 20-fold molar excess over integrase. We conclude that these proteins are unlikely to play an important role in promoting concerted integration.

We were surprised that several hundred base pairs of viral DNA substrates are required for maximal efficiency of concerted integration, because only 20 bp of substrates efficiently carry out half-site integration and less than 20 bp of the terminal sequence are protected by integrase in footprinting experiments (17). Furthermore, a very large multimer of integrase would be required to interact with several hundred base pairs of DNA. We speculated that nonspecific binding of integrase along DNA may facilitate the formation or stabilization of a specific complex of an integrase multimer with the terminal viral DNA sequence. We note that the integration efficiency of longer viral DNA substrates is more robust at higher salt concentrations compared with shorter substrates (data not shown). This is consistent with the idea that multiple interactions along the DNA stabilize the interaction of integrase with the viral DNA substrate.

HIV-1 integrase exists as monomers, dimers, and tetramers in solution (29, 38–40), but the multimeric species that mediates concerted integration is unknown. Although the functional relevance of the interfaces observed in the crystal and solution structures of the individual integrase domains is uncertain, the extensive dimer interface of the catalytic domain is conserved in all of the structures determined to date (41–49). It therefore seems likely that this interface will also be present in the active complex. However, the spacing and location of the two active sites within this dimer suggest that this pair of active sites would be incapable of performing concerted integration. Because the sites of joining the two viral DNA ends to target DNA are separated by 5 bp, the nucleoprotein complex that carries out concerted integration is expected to include a pair of active sites with a similar spacing. However, the two active sites in the catalytic domain dimer are inappropriately positioned to span the two sites of joining to target DNA. A higher order multimer is likely required to juxtapose a pair of active sites with the correct spacing. The profound differential effects of mutation of Trp-235 on half-site *versus* concerted integration implicates this residue and the C-terminal domain in an interface that is required for concerted integration but not for the half-site reaction.

Despite progress in understanding the biochemistry of integration and the structures of the individual domains of integrase, remarkably little is known about the nucleoprotein complex that mediates concerted integration. Elucidation of the organization of this complex will be necessary to understand how the integration of pairs of viral ends is coordinated. Furthermore, because inhibitors of integrase must recognize this complex in the cell, knowledge of its structure will be necessary to understand their mechanism of action.

## Supplementary Material

supplementary Material

## Footnotes

1 The abbreviations used are: HIV, human immunodeficiency virus; PIC, preintegration complex; LTR, long terminal repeat; MES, 4-morpholineethanesulfonic acid; IN, integrase; NC, nucleocapsid.
