# Supplementary material for: Processing of Viral DNA Ends Channels the HIV-1 Integration Reaction to Concerted Integration
Source: J Biol Chem. Author manuscript; Available in PMC 2022 Jan 8. (PMC8742673; doi:10.1074/jbc.M505367200)
Supplement: supplementary Material [file NIHMS1766426-supplement-supplementary_Material.pdf]

Supplementary Figure 1

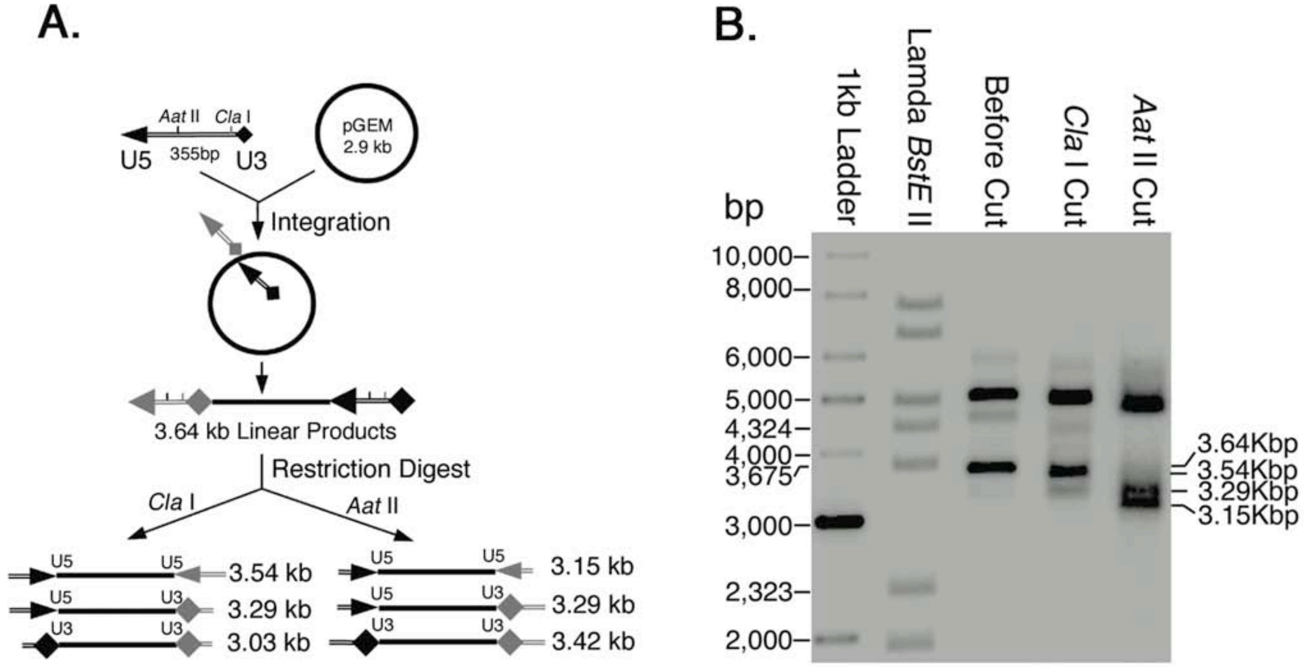

Fig 1. Restriction analysis of integration products. 355 bp blunt end U3/U5 DNA and supercoiled plasmid DNA (pGEM9) were used as the donor and target, respectively. Integration products were purified by phenol-chloroform-isoamyl alcohol extraction and then chloroform only, followed by ethanol precipitation. Purified integration products were digested with Cla I or Aat II and separated by electrophoresis in a 0.8% TBE agarose gel. (A) Schematic of the possible forms of concerted integration products after digestion of the 3.64 kb linear product by Cla I or Aat II. (B) Autoradiogram of the digested integration products. The products of Cla I digestion are 3.54 kb and 3.29 kb for integration of a pair of U5 ends and a U5/U3 end pair, respectively. The products of Aat II digestion are 3.15 kb and 3.29 kb for integration of a pair of U5 ends and a U5/U3 end pair, respectively. The upper band is the half-site product and little change in electrophoretic mobility is expected upon digestion because it remains circular.

Supplementary Table 1

Sites of Integration of the U5 Donors Into Acceptor DNA

Sequence of donor-acceptor junction	Size of duplication/deletion	position of integration on plasmid
**pTRG plasmid DNA as acceptor**		
AGCAggttg------ggttgTGCT	5bp duplication	3018-3014
AGCAggtct------tgatcTGCT	29bp duplication	2992-3020
AGCAgcacc------gcaccTGCT	5bp duplication	2944-2940
AGCAgcgca------gcgcaTGCT	5bp duplication	2943-2937
AGCAcgttg------gtgcgTGCT	17bp deletion	2922-2938
AGCAaccct------accctTGCT	5bp duplication	2604-2600
AGCActcca------ctccaTGCT	5bp duplication	2410-2414
AGCAcagct------cagctTGCT	5bp duplication	2359-2399
AGCAgcggc------gcggcTGCT	5bp duplication	1720-1724
AGCAttccc------ttcccTGCT	5bp duplication	1684-1688
AGCAgccca------gcccaTGCT	5bp duplication	1613-1617
AGCAccgaa------gggtcTGCT	34bp deletion	608-565
AGCAggcgg------ggcggTGCT	5bp duplication	595-591
AGCAgagca------gagcaTGCT	5bp duplication	458-462
**pGPS3 plasmid DNA as acceptor**		
AGCAgatgg------gatggTGCT	5bp duplication	3736-3732
AGCAcgtaa------gggagTGCT	160 bp deletion	3082-2921
AGCAggttg------ggttgTGCT	5bp duplication	3017-3013
AGCAaatac------aatacTGCT	5bp duplication	2044-2048
AGCAgcgcc------gcgccTGCT	5bp duplication	1823-1827
AGCActaca------tttgcTGCT	18bp deletion	1506-1487
AGCAgtggg------gtgggTGCT	5bp duplication	1340-1336
AGCAggaag------ggaagTGCT	5bp duplication	608-612
AGCAtccga------tccgaTGCT	5bp duplication	562-567
AGCAgctgc------gctgcTGCT	5bp duplication	217-221
AGCAgagat------gagatTGCT	5bp duplication	61-57

355bp U3/U5 DNA and pTRG (stratagene) or pGPS3 (New England Biolabs) plasmid DNA were used as the donor and target in the integration reaction. Products were purified and served as template for PCR. Oligonucleotide primer P5 (5'-GTCAGTGTGGAAAATCTCTAGC) was used for amplification of the two-ended U5/U5 integration product with 2.5 U Platinum Pfx DNA Polymerase (Invitrogen) and 0.30μM primers in 50μl reaction volumes. P5 was complementary to the LTR sequences at each end of the linear two-ended products and served as both upstream and downstream PCR primers. The PCR was performed for 1 cycle of 95°C for 1 min, and was followed 30 cycles at 95°C for 1min, 56°C for 1min and 72°C for 4.5min, then extended at 72°C for 7 min. The PCR products were purified from an 0.8% TBE agarose gel and cloned using a Zero Blunt PCR Cloning Kit (Invitrogen). Recombinants containing PCR products were scored as two-end integrants. Plasmids were sequenced by M13 forward and reverse primers to analyze the junctions between the LTR and target DNA.
